# The Impact of Hydroxyapatite Sintering Temperature on Its Microstructural, Mechanical, and Biological Properties

**DOI:** 10.3390/ijms24065083

**Published:** 2023-03-07

**Authors:** Marta Trzaskowska, Vladyslav Vivcharenko, Agata Przekora

**Affiliations:** Independent Unit of Tissue Engineering and Regenerative Medicine, Medical University of Lublin, Chodzki 1, 20-093 Lublin, Poland

**Keywords:** bioceramics, biocompatibility, cytotoxicity, bioactivity, mechanical properties, bioabsorbability

## Abstract

Hydroxyapatite (HA), the principal mineral of bone tissue, can be fabricated as an artificial calcium phosphate (CaP) ceramic and potentially used as bioceramic material for bone defect treatment. Nevertheless, the production method (including the applied sintering temperature) of synthetic hydroxyapatite directly affects its basic properties, such as its microstructure, mechanical parameters, bioabsorbability, and osteoconductivity, and in turn influences its biomedical potential as an implantable biomaterial. The wide application of HA in regenerative medicine makes it necessary to explain the validity of the selection of the sintering temperature. The main emphasis of this article is on the description and summarization of the key features of HA depending on the applied sintering temperature during the synthesis process. The review is mainly focused on the dependence between the HA sintering temperature and its microstructural features, mechanical properties, biodegradability/bioabsorbability, bioactivity, and biocompatibility.

## 1. Calcium Phosphate Bioceramics—Biomaterial Widely Used for Bone Tissue Regeneration

Bone is a tissue with a high capacity for self-repair, allowing the reconstruction of a damaged fragment without involvement of scar tissue. Despite this fact, some clinical cases due to external factors or accompanying diseases require surgical intervention [1,2,3]. In orthopedic surgeries aiming to regenerate serious bone fracture or defects, autologous grafts are the first choice due to the highest probability of successful regeneration. Nevertheless, the harvesting of autologous tissue has some limitations: the small amount of healthy tissue that can be safely used for this purpose, the burden on the body and the cost of the potential additional surgery, and complications at the bone harvesting site [4,5,6,7]. The development of synthetic calcium phosphate (CaP) bioceramics emerges as a distinct strategy that can overcome the limitations related to autologous tissue collection [8]. In the scientific literature, the predominant types of CaPs used are: tricalcium phosphates (TCPs, Ca2(PO4)3), hydroxyapatite (HA, Ca10(OH)2(PO4)6), and biphasic calcium phosphates (BCPs) [9]. Some key differences observed between the listed CaP types determine the frequency of their use in the case of bone regeneration [10]. The optimal CaP-based biomaterial used in the regeneration of bone tissue should be characterized by biocompatibility, osteoconductivity (the ability to promote osteoblast adhesion, proliferation, and differentiation), and suitable mechanical parameters (compressive strength and Young’s modulus) close to the human bone. Additionally, the high porosity and high specific surface area (SSA) of HA are essential for its biodegradability (thus bioabsorbability in a living organism), high bioactivity (ability to form an apatite layer on the HA surface), and good osseointegration of the material with the host tissue [8,11,12]. 

Synthetic CaP bioceramics are obtained from chemical reagents by controlled synthetic processes that allows one to obtain the material with desired microstructural and physicochemical properties in order to fit the specific requirements of different clinical cases [9,13,14]. When the bone regeneration is impaired, the osteogenic potential of CaP-based grafts can be enhanced by combining them with drugs, growth factors, or cell-based therapies [15,16]. Although there is a large variety of CaP-based materials for bone regeneration on the market, including granules, powders, blocks, cements, scaffolds, etc. [15,17,18], CaP granules are still frequently used in orthopedics and maxillofacial surgeries to fill bone defects and stimulate the regeneration process [8]. The current article was prepared mainly according to the scientific contributions in the ScienceDirect and Web of Science databases using the keywords: hydroxyapatite; sintering; bioceramics; biocompatibility; cytotoxicity; bioactivity; biodegradation; microstructure; mechanical properties. The database search was double-checked and supported with Google Scholar.

## 2. Synthesis of Sintered Hydroxyapatite

**Figure 1:**
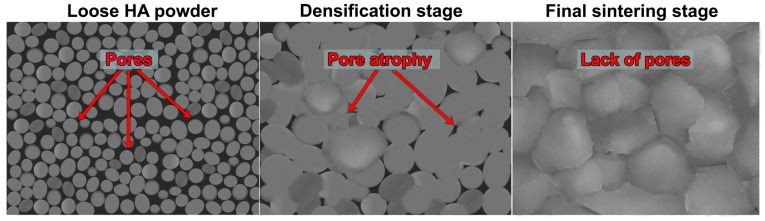
Schematic representation of the influence of the sintering process stages on changes in pore structure of hydroxyapatite (prepared based on the information found in [29,30,31]).

The functional and structural similarity of HA to a mineral composition that is present in teeth and bones explain its priority over other CaPs [19,20]. Historically, granules of dense hydroxyapatite (HA) obtained by sintering at high temperature (≥900 °C) were the first bioceramics used as synthetic bone grafts [8]. Particular interest in HA results from its high similarity to the mineral part of the bone matrix, its biocompatibility, and its ability to create a strong bond with bone tissue without the participation of fibrous tissue [21]. Traditionally, in order to obtain a material with a compact structure, hydroxyapatite powder is densified as a result of the action of one or a combination of the following factors: temperature, pressure, and time of exposure to high temperature [22]. This process, called sintering, is a very important stage in the production of ceramics, since it affects the final microstructure of the material, i.e., the shape and size of grains, degree of porosity, and pore size, as well as the chemical composition and crystal structure. These parameters, in turn, affect the mechanical strength and biological properties of ceramics [18,22,23,24]. Three main stages can be distinguished in the sintering process: 

1. Neck growth stage: The powder particles are still units and are not physically bound. Tensile stresses resulting from surface tension stabilize the surface of the powder particles between adjacent grains in the contact plane. So-called “necks” start to form between the powder particles, joining them together (the formation of necks between the grains is powered by the high surface free energy of the system) [25]; 

2. Material densification stage: Independent powder grains begin to lose their individual boundaries. The boundaries of the enlarged grains define the pores of the material. As the grain grows, the number of pores steadily decreases [26];

3. Closing of the last pore spaces stage: The remaining residual pores become more and more spherical and are no longer open to the outside. Non-diffused gases are isolated in closed pores. The entire sintering process, during which a clear densification of the material is observed, covers at least the first two stages (Figure 1) [22,27,28].

**Figure 2:**
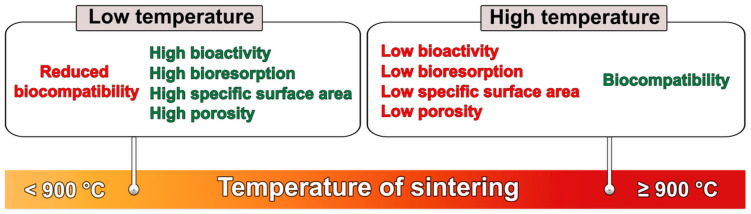
The impact of HA sintering temperature on its basic properties.

The rate of sintering depends, among other factors, on the temperature, type of material, and powder particle size. The binding of molecules results in greater mechanical strength and lower system energy [29]. Importantly, unlike HA sintered at low temperature, the ceramics produced by high-temperature sintering are highly biocompatible (Figure 2) [32,33]. 

Despite very good tolerance by the organism, HA sintered at high temperature (≥900 °C) is not an ideal material. This type of ceramic is characterized by low porosity and low specific surface area (SSA), is not biodegradable, has low bioactivity, and is too brittle to be applied in load-bearing implantation areas. Importantly, it was observed that osteoclasts are unable to dissolve ‘super stable’, stoichiometric, and highly crystalline HA sintered at high temperature, which cannot undergo bone remodeling and is present in the bone defect for years, hindering osseointegration [8,34,35]. Improvement of HA bioabsorbability can be achieved by lowering the temperature during the material sintering process, which results in the increase in porosity and SSA of the bioceramics. HA of higher SSA (approx. 10–60 m^2^/g), compared to HA sintered at high temperature (approx. 2–5 m^2^/g), is often produced at a temperature in the range of 200–800 °C. As a consequence, the resultant HA has better bioabsorbability and higher bioactivity. Unfortunately, it has been reported that HA synthesized using low sintering temperatures frequently exhibits a cytotoxic effect on eukaryotic cells in vitro due to high ionic reactivity [34,35,36,37,38]. 

This review discusses the most important problems encountered by scientists investigating in vitro and in vivo materials made of hydroxyapatite sintered at high (≥900 °C) and low (<900 °C) temperatures. The review is divided into five sections describing the impact of the sintering temperature of HA-based biomaterials on their mechanical properties, microstructural features, biodegradability/bioabsorbability, bioactivity, and biocompatibility. The article was prepared according to the scientific contributions in the ScienceDirect and Web of Science databases (supported with Google Scholar searching). 

## 3. Mechanical Properties of Sintered HA 

The mechanical properties of the implantable material, e.g., flexural strength, compressive strength, Young’s modulus, and tensile strength, should be as similar as possible to those of bone tissue [39,40,41,42]. Appropriate mechanical strength and preservation of the structure after implantation is particularly important in the case of supporting tissue, which is bone [43]. It is expected that the mechanical strength of the biomaterial will remain sufficient until regeneration is completed, and new tissue will be able to provide adequate support [39]. The mechanical properties of HA produced by sintering are affected by grain size, connections between grains, pore shape, and material density. These parameters can be adjusted by changing the sintering temperature [44]. 

According to the scientific literature, an increase in sintering temperature resulted in improvement of compressive strength and density, but simultaneously reduced the HA material’s porosity and elastic deformation according to Young’s modulus values (Table 1 and Table in Section 4) [24,44,45,46]. 

Additionally, the use of HA sintered at high temperature as an implantable material is restricted, among other reasons, due to its high brittleness. Dense HA is particularly susceptible to the slow spreading of small defects on its surface, known as slow crack growth. This process is the cause of delayed damage of the ceramic when the size of the crack grows to a critical value. This is one of the main limitations of the use of CaP ceramics [47]. Benaqqa et al. [48] aged a material made of HA sintered at 1200 °C in a 37 °C water bath for 6 weeks, which is the approximate time required for bone injury to heal in vivo. After this time, a decrease in mechanical properties and density was observed (decrease in density from 98% to 95%). The sintered HA showed high sensitivity to slow crack growth caused by water stress corrosion cracking after aging in water. The same authors confirmed that slow crack growth takes place in the aqueous environment at extremely low stress intensity factors (0.4 MPa√m), which excludes the use of HA in places subjecting it to high loads. Prolonged exposure to a wet environment further increases the sensitivity of HA to cracking. Moreover, the use of temperatures above 1200 °C during the sintering process leads to the deterioration of this parameter due to microcracks and intercrystalline cracking [49]. Similar results were obtained by De With et al. [50]. It was shown that the strength and fracture toughness of the dense, sintered HA measured in wet conditions was more than half lower compared to the values of these parameters measured in dry conditions. The authors concluded that the mechanical strength of this type of material is likely to be sufficient only for short-term loads due to the significant loss of strength over time, especially in the wet conditions. The wear of HA sintered at 1200 °C was also investigated by making a scratch in water under a normal load of 1 to 10 N. The application of a 1–6 N load resulted in a more plastic wear pattern and the appearance of a small number of cracks. However, as a result of the use of a load from 7 to 10 N, a brittle type of surface destruction was observed, which was indicated by numerous damages such as: cracks, stretching, and the formation of chevrons in the space adjacent to the crack [51]. The brittleness of HA sintered at high temperature was also confirmed by in vivo studies. De Lange et al. [52] implanted in the mandibles of dogs dental implants that were made of dense, sintered HA. The implants were solid or hollow and of cylindrical shape. Hollow scaffolds were placed between two titanium caps for initial compression. The implants were subjected to physiological loads for a period of 6 months to 5 years. Six months after implantation, it was observed that 29% of the solid HA implants had fractured in the cervical part of the scaffold, while the root part was still immobilized in the alveolar ridge. Within a year and a half, that number had increased to 76% of implantations failing. However, most of the pre-compressed scaffolds on the titanium caps did not break and remained stably immobilized in the jaw during the study. On the other hand, Pauchiu et al. [34] evaluated the influence of the environment and sintering temperature in the range of 900–1350 °C on the density, mechanical hardness (Knoop test), and bending strength of HA. The analyzed mechanical parameters increased proportionally to the temperature of sintering in air conditions until the application of 1150 °C, above which a decrease was noted. The authors indicated that the decomposition of HA into tricalcium phosphate (TCP) and tetracalcium phosphate (TTCP) was responsible for this tendency. In the case of sintering under vacuum conditions in the same temperature range, the mechanical parameters of HA had inferior values. Sintering in a humid environment resulted in an increase in mechanical properties until the temperature of 1300 °C was reached. However, below the temperature of 1200 °C, the mechanical parameters of HA sintered in moisture conditions had lower values compared to sintering in air conditions at the appropriate temperatures. 

## 4. Microstructural Properties of Sintered HA

**Figure 3:**
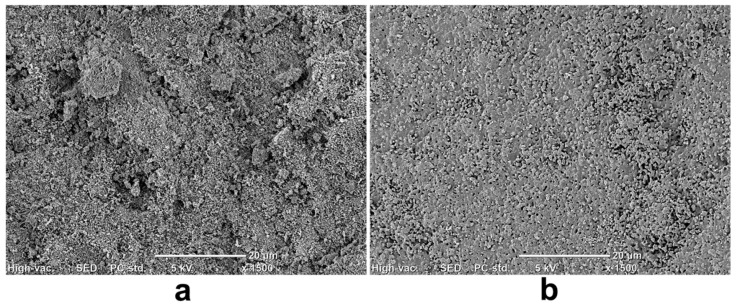
Scanning electron microscope images presenting microstructure of hydroxyapatite (HA) dependent on the sintering temperature: (**a**) rough and porous microstructure of HA sintered at 800 °C; (**b**) dense and non-porous microstructure of HA sintered at 1250 °C.

Adjustment of the implant microstructure during its production is extremely significant since it affects biocompatibility, biodegradation, bioactivity, and mechanical properties. As part of the microstructure of the material, the SSA, grain size, surface topography, and porosity (size and quantity) are verified. Both the presence of micropores and macropores are very important for bone function [53]. Considering CaP-based bone implants, macropores create a surface in ceramics essential for cell adhesion, proliferation, and tissue ingrowth, whereas the presence of micropores increases SSA, affecting the material’s ionic reactivity, bioactivity, and ability to absorb liquids and adsorb proteins [54]. Moreover, the increase in HA microporosity causes a rise in the surface area in contact with the body fluids, which significantly accelerates the biodegradation process and the deposition of apatite on the surface of the material (bioactivity) [32,54]. However, a balance between the content of pores in the material and its mechanical properties is necessary. The use of high sintering temperature results in increased strength of the sintered HA, but at the same time it reduces porosity and SSA (Figure 3). The decrease in the porosity and SSA of CaP ceramics is associated with less effective bioactivity and osseointegration of the material, which may hinder the implantation process [44,55]. The dependence between sintering temperature and HA microstructural properties (porosity, SSA, and density) is presented in Table 2.

Prokopiev and Sevostianov [44] investigated the effect of sintering temperature (in the range of 1140–1340 °C) on the microstructure and mechanical properties of hydroxyapatite. The tests showed that the use of a temperature in the range of 1140–1220 °C during the sintering process resulted in a strong density of HA, while above the temperature of 1220 °C no further changes in density were observed. It was also noted that in the temperature range of 1280–1340 °C, the number of pores decreased significantly with increasing temperature, while their size increased (the overall porosity did not change). In addition, the pores of HA sintered in the temperature range of 1140–1280 °C had a flattened shape. Above the temperature of 1280 °C, their shape changed to a more round one as a result of a clear diffusion of matter in the vicinity of sharp pores. Moreover, it was demonstrated that grain size increased with increasing temperature. For the lowest and highest temperature used, the grain size was 2.3 µm and 3.5 µm, respectively. A similar tendency was shown in the study of Prokopiev et al. [56]. It was noticed that the pores of an HA sample sintered at 1350 °C had a more round shape compared to the HA pores sintered at lower temperatures. It was also suggested that an increase in the sintering temperature could have closed some of the microcracks in the material. In another paper, Muralithran and Rames [30] reported that an HA sample sintered in the temperature range of 1000 to 1450 °C reached its maximum density when using temperatures of ≥1200 °C. The SEM imaging showed the presence of large pores in HA sintered at temperatures below 1200 °C. In materials sintered above 1200 °C, the content of pores was below 1%. The few pores in these materials occurred mainly at grain boundaries. The increase in grain size was slow until reaching the temperature of 1250 °C. Above 1250 °C, the grain growth was significant (2.03 µm for 1250 °C and 12.26 µm for 1400 °C). In another study, Malina et al. [18] evaluated the sintering temperature range of 800 to 1400 °C. According to the presented results, temperature rise significantly increased the apparent density and decreased the porosity of the tested sintered materials. Obada et al. [24] analyzed the influence of the sintering temperature (900, 1000, and 1100 °C) on HA porosity. The reduction in the materials’ porosity was related to increasing sintering temperature and varied from 51.7 ± 0.06 to 46.9 ± 0.10%. According to Oktar et al. [46], HA compacts’ densities were affected by the selected sintering temperature. The reduction in the samples’ density was observed with the application of reduced sintering temperature. The highest density (3.03 g/cm^3^) was obtained for HA sintered at 1300 °C and the lowest (2.15 g/cm^3^) for HA sintered at 1000 °C. In the case of SSA evaluation after the HA sintering process, Bailliez, and Nzihou [57] revealed that with the increase in sintering temperature from 550 °C to 1000 °C, the SSA of the sample decreased from 28 to 3 m^2^ g^−1^, respectively. No significant SSA reduction was observed above 1000 °C. The reduction in SSA with the increase in the HA sintering temperature was also proved by Wang et al. [58]. According to the research, a significant increase in the SSA took place already at 800 °C compared to higher temperatures. The SSA reduction at higher sintering temperatures was mainly caused by the aggregation of small HA particles. A significant decrease in SSA as a result of increasing the sintering temperature was also presented in another study [60] where the increase in temperature from 1000 to 1400 °C resulted in a great SSA decrease. Similar results were obtained by Radin and Ducheyne [59] where in the case of HA sintered at a low temperature of 200 °C, the SSA of HA was approximately tenfold higher (45.2 g/cm^3^) compared to HA sintered at 900 °C (4.7 g/cm^3^). 

When characterizing the porosity of a HA-based biomaterials, parameters such as the degree of porosity, pore size, and interconnectivity between the pores should be primarily taken into account. SEM analysis provides a qualitative assessment of pore size and wall thickness; however, it should be noted that the measurement is carried out only on the surface of the sample [61]. In turn, mercury intrusion porosimetry is a well-known and often used method for the quantitative evaluation of the open porosity of HA-based biomaterials, in the pore size range of 0.0018 to 400 μm. It enables the examination of properties such as pore tortuosity or cumulative surface area to pore size. The disadvantage of this technique is the lack of information about the interconnectivity of the pores and the closed porosity [61,62]. Another technique for measuring pore volume and pore size distributions is gas adsorption. In addition, this procedure enables the determination of pore shapes and the areas of mesopores and micropores [61]. Micro CT is a relatively new method of porosity analysis, but it has gained a lot of supporters due to its many advantages over other techniques. It provides the most comprehensive evaluation of porous biomaterials in terms of measuring pore size and interpore connections. Furthermore, thanks to this method, it is possible to visualize closed and interconnected pores, while the tested material is not damaged during the procedure and can be used for further research [63]. Nevertheless, it is not a suitable technique for porosity evaluation of powders and granules. It is also not recommended for biomaterials characterized primarily by microporosity.

## 5. HA Biodegradability Dependent on the Sintering Temperature 

Biodegradability/bioabsorbability is another essential property of bone tissue biomaterial [64,65,66]. In order to provide suitable space for the newly formed bone tissue, the material should degrade over time in vivo. The rate of bioresorption should be appropriately adjusted to the rate of growth of new bone in the process of healing [67,68,69]. The products resulting from the biodegradation should not have a toxic effect on the host cells, and the body should be able to metabolize and eliminate them [43]. Materials produced from inorganic compounds can be biodegraded by two processes: dissolution through physicochemical reactions and bioresorption through biological processes. Bioresorption takes place thanks to the cellular activity, especially of osteoclasts and macrophages, through acidification of the environment and enzymatic reactions [68]. The biodegradation process depends on many variables. Microstructural and physical factors include: the form of HA (e.g., granules, scaffold, and grain size), SSA, porosity, and crystallinity. The following factors are known to stimulate the biodegradation process: small crystal sizes, large amounts of crystal imperfections, and a high degree of porosity. Chemical factors include: the ingredients and ionic substitutions of HA, while biological factors include: the pH of the microenvironment, infection, bone contact surface, and bone type [70,71]. The content of macro- and micropores in the material plays a key role in the dissolution process of calcium phosphate ceramics. The rate of decomposition of the material increases proportionally to the increase in the surface in contact with the environment, since more exchanges, e.g., of ions, can take place [72]. The biodegradation and bioresorption ability of HA materials sintered in a wide range of temperatures is presented in Table 3.

Ducheyne et al. [83] investigated the influence of the crystallinity and stoichiometry of calcium phosphate ceramics on the rate of its biodegradation in vitro in a Tris buffer solution (pH = 7.3) without calcium and phosphate ions. The following materials were tested: calcium-deficient HA, β-tricalcium phosphate, α-tricalcium phosphate, oxyhydroxyapatite, stoichiometric HA, and tetracalcium phosphate. The analyses showed that among all tested biomaterials the slowest degradation was recorded in the case of dense, well-crystallized, stoichiometric HA sintered at 900 °C. The influence of crystallinity and the number of ionic substitutions on the solubility of the ceramics was also emphasized by Fulmer et al. [73]. Commercially available, sintered hydroxyapatite (Calcitite) characterized by high crystallinity and a lack of ionic substitutions was the least soluble among the tested apatite materials with reduced crystallinity and carbonate substitutions. Stastny et al. [74] also observed low solubility of HA in vitro. After 14 days of incubation in a solution with an acidic pH of 5.5 (McIlvaine buffer), the structure of the scaffold made of HA sintered at 1250 °C was almost unchanged. The mass loss of the HA material was only 3%, and the compressive strength of the HA samples did not change during the experiment. Slow biodegradation of HA materials sintered at high temperatures was also reported using in vivo models. Lu et al. [71] analyzed the biodegradation of an implant made of HA sintered at 1270 °C placed in the condyle of the tibia or femur of rabbits. Studies showed that during the 24-week implantation period, the HA material degraded to a very small extent. The shape and architecture of the implant did not change significantly. However, HA ceramics were characterized by high biocompatibility. No rejection by the body and no necrosis or infection were observed. Similar research results were demonstrated by Klein et al. [75]. Scientists placed cylinder-shaped implants made of calcium phosphate (sintered HA or P-whitlockite) in the tibia of rabbits. No inflammatory reaction resulting from the presence of the implants was observed during the experiment. In contrast to the sintered P-whitlockite material, which was biodegraded to a degree depending on the degree of porosity, the material made of sintered HA showed no signs of biodegradation during the experiment (9 months). It should be noted that the hydroxyapatite material was sintered using a temperature of 1300 °C or 1100 °C and showed a porosity similar to that of the P-whitlockite material. This confirms the assumption that porosity is a necessary factor influencing biodegradation, but not the only one, since the materials used in the tests also differed in terms of Ca/P ratio and crystallinity. Considering the bioresorption of HA, the scientists likewise did not notice any major changes on the surface of the sintered HA. Imaizumi et al. [76] compared the biodegradation of granules made of HA sintered at 1150 °C and octacalcium phosphate (OCP) after 12 weeks from implantation at the site of rabbit femur defects. They noticed that HA remained almost untouched. After 8 weeks of implantation, a larger area occupied by multinucleated giant cells (also with the phenotype: tartrate-resistant acid phosphatase (TRAP)-positive, indicating the presence of osteoclasts) was observed in the case of OCP material compared with the HA surface. Yamada et al. [77] assessed the effect of the solubility of materials belonging to CaP ceramics on their bioresorption by osteoclasts. HA sintered at 1000 °C was the least soluble CaP material used in the study. SEM images of HA after two days of culture of neonatal rabbit bone-resorbing cells on its surface showed no signs of bioresorption. Interestingly, the bioresorption on the surface of more soluble material used in the experiment (β-TCP) was not greater than on the material consisting of a mixture of HA and β-TCP. Thus, the intensity of bioresorption can only be proportional to the solubility of the material to a certain extent. Doi et al. [78] observed that osteoclasts, after a two-day incubation on the surface of HA sintered at 1200 °C, adhered in a similar way as to the surface of bone or resorbable ceramics. However, no traces of the bioresorption in the form of pits or lacunae were detected on the HA surface. The level of calcium and phosphate ions in the culture medium was also unchanged. In another study, Hasegawa et al. [79] implanted porous, cylindrical implants made of HA sintered at 1200 °C in rabbits’ knee defects. The experiment was carried out for a maximum of 12 months. No osteoclast-like cells were observed on the surface of HA. Moreover, the porosity of the HA samples did not change during the experiment, which proved the lack of biodegradation. The authors also indicated that the bioresorption may be influenced by the size of the HA crystals (0.1 μm) [79]. Different results were presented in the article by Nakamura et al. [84]. They cultured osteoclasts differentiated from peripheral mononuclear blood cells (PBMC) on the surface of ceramic materials for 15–18 days. The cells were able to differentiate by the addition of the receptor activator of nuclear factor-kappa B ligand (RANKL) and macrophage colony-stimulating factor (M-CSF) to the culture medium. The results of the experiments showed that on the surface of HA calcined at 800 °C and then sintered at 1250 °C, resorption pits were formed; however, they were indistinct, and their depth was much smaller than on the surface of the carbonated HA. In addition, on the HA surface, PBMCs differentiated to a lesser extent into TRAP-positive giant multinucleated cells. During bioresorption, structures called actin rings are formed in activated osteoclasts, which are responsible for creating a tightly closed space between the cell membrane and the substrate. Inside these structures, a resorption cavity is formed, which is the site of secretion of protons and proteases by osteoclasts. Nakamura et al. [84] demonstrated that on the HA surface, the actin rings in the osteoclasts were thin and had large diameters, in contrast to the thick actin rings with small diameters observed in the osteoclasts on the carbonated HA surface and bone sections. During the research, vinculin was also imaged. In the case of the HA samples, vinculin was dispersed in the central part of the cells, while in the bone and carbonated HA samples it was located in the same place as the actin rings. Takeshita et al. [80] also observed multinucleated giant cells on the surface of HA sintered at 200 °C (HA200) 1–3 weeks after implantation in the mandible of a rat. The cells on the HA200 material had a large, polygonal area of cytoplasm, three to ten nuclei, large mitochondria, a well-developed Golgi complex, and numerous vesicles. Importantly, these cells also had ruffled borders, containing clear zones and a folded cytoplasmic membrane. Ruffled borders are considered to be the most characteristic feature of functional osteoclasts. A lower number of multinucleated giant cells was observed on the surface of HA sintered at 1250 °C (HA1250) than on HA200 material. Cells on the surface of HA1250 showed a similar arrangement and development of organelles as those on the surface of HA200. However, the cells on the surface of HA1250 were observed to form an undeveloped ruffled-border-like structure with clear zones only. In the research conducted by Goto et al. [81], blocks of synthetic HA sintered at 900 °C were implanted into a patient’s tumor bone defect. After 40 months, the HA block became smaller, indicating its biodegradation. After 79 months, the HA block almost disappeared and was replaced with newly formed bone. Similar results were obtained by Winter et al. [82] where HA material sintered at 900 °C was used as the midshaft femoral implant in a rat model. Nests of macrophages were observed between the HA implant surface and new bone tissue a month after implantation. After 6 months, full activity of the biodegradation process was observed. A great amount of HA granular material was phagocytosed and transported into the marrow cavity.

## 6. HA Bioactivity Dependent on the Sintering Temperature

In vitro studies showed that materials with high solubility are also characterized by high bioactivity [85]. The bioactivity of a material means its ability to form a hydroxycarbonate apatite layer on the surface after contact with certain body fluids [86,87,88]. This is a very important property since the precipitated apatite forms a layer connecting the material with the host bone tissue, contributing to the osseointegration process. In addition to anchoring in the tissue, the bioactive material increases the adhesion of osteoblasts, stimulates the differentiation of mesenchymal stem cells and their enzymatic activity, and enhances the formation of new blood vessels [89,90]. In vivo formation of biological apatite can be recreated to some extent in vitro by incubation materials in a simulated body fluid (SBF) solution that has ion concentrations similar to those found in body fluids [85,91,92]. The apatite layer deposition on the surface of HA depending on its sintering temperature is presented in Table 4.

Kim et al. [31] revealed that HA sintered at a low (800 °C) and high (1200 °C) temperature had the ability to form an apatite layer on the surface as a result of the same process. Due to the presence of negative hydroxyl and phosphate groups on the surface, the HA in the SBF solution was negatively charged and was therefore able to interact with the positively charged calcium ions in the fluid to create a calcium-rich apatite. Calcium-dominant apatite had a positive surface charge and interacted with negatively charged phosphate ions in the environment to create calcium-poor apatite. This apatite was stabilized by the crystallization process, resulting in the form of apatite present in the bones. In the next stage, apatite was spontaneously formed by incorporating further calcium and phosphate ions as well as smaller ions such as magnesium, sodium, or carbonate, becoming more and more similar to the mineral part of the bone. However, it was demonstrated that this process was slower in vitro in the case of HA sintered at a higher temperature (1200 °C) compared to HA sintered at 800 °C. The time needed for the formation of calcium-rich, calcium-poor, and crystallized apatite on the surface of HA sintered at 800 °C and 1200 °C was 3, 6, and 9 h and 6, 9, and 12 h, respectively. The authors suggested that the lower negative charge on the surface of ceramics sintered at 1200 °C at the beginning of the process was responsible for this effect due to the smaller amount of hydroxyl and phosphate groups available on the surface. Mezahi et al. [93] also noticed that the precipitation of a new layer of apatite on the surface of HA sintered at 800 °C was faster (after 7 days of incubation in SBF) compared to HA sintered at 1200 °C (after 30 days of incubation). In addition, Shi et al. [97] revealed a dependency between the bioactivity of coatings made of HA and the SSA of the material and its crystallinity in vitro. It was demonstrated that the placement of HA sintered at 600 °C and 700 °C in the SBF resulted in an immediate uptake of calcium ions. On the other hand, HA sintered at 800 °C and 900 °C had been partially dissolved before precipitation occurred. The authors indicated that small HA crystals formed at low-temperature sintering had high surface energy, and thus a lower driving force or supersaturation was needed for their nucleation. A high degree of crystallinity negatively affected the bioactivity process, since a surface of this type required a high driving force to precipitate a new apatite layer. Thus, well-crystallized surfaces with low energy (HA sintered at 800 °C and 900 °C) must initially partially dissolve, as a result of which HA will reach the appropriate supersaturation to induce the precipitation of a new phase. A linear relationship between the rate of new apatite formation and the SSA was also demonstrated. In another study, Radin and Ducheyne [83] investigated the formation of carbonate-containing HA on the surface of materials belonging to CaP ceramics (stoichiometric hydroxyapatite s-HA, oxyhydroxyapatite, Ca-deficient hydroxyapatites, β- and α-tricalcium phosphate, and tetracalcium phosphate) incubated in a simulated physiologic solution (SPS). Among all tested materials, a new apatite layer was formed the fastest on the surface of HA with poor crystallization (HA sintered at 200 °C and 600 °C), and then with good crystallization (HA sintered at 900 °C and 1100 °C). However, it should be noted that during the experiment no apatite containing carbonate was formed on the surface of well-crystallized HA, but only intermediate forms of apatite. The authors suggested that the structure and composition of the material had the greatest influence on the formation of apatite. Nevertheless, there was no clear correlation between the degradation rate of the material and bioactivity. The same authors also compared the reactivity of porous ceramics (HA) with dense ceramics (stoichiometric HA sintered at 200 °C and 900 °C) [59]. In this study, HA sintered at 200 °C was defined as dense, poorly crystallized HA, while HA sintered at 900 °C was defined as dense and well-crystallized; however, the porosity of both materials was not investigated. The research revealed that only in the case of HA sintered at 200 °C was the immediate formation of apatite observed. The time needed to induce apatite formation for porous HA and dense HA sintered at 900 °C was the same (60 min), but the amount of calcium ions captured in the solution was higher for porous HA. The authors indicated that the induction time of apatite precipitation of the HA sintered at 200 °C and 900 °C and porous HA materials was probably affected by the size of the SSA, which was 45.2 m^2^/g, 4.7 m^2^/g, and 4.0 m^2^/g, respectively. Furthermore, Cho et al. [94] showed that 7 days after immersion of HA sintered at 1100 °C in SBF, the material was not able to form a new layer of hydroxycarbonate apatite on its surface. During the experiment, no changes in the pH or concentration of calcium and phosphate ions in the SBF solution were noted. Chloride-substituted HA, on the other hand, was characterized by increasing bioactivity as the amount of chloride substitutions increased. It was demonstrated that the presence of chloride substitutions also improved the solubility of HA. A faster release of the component ions of the material increased the supersaturation in the SBF solution, which resulted in the increased bioactivity. In another scientific report, it was also shown that doping HA with hexavalent tungsten improved the bioactivity of sintered HA. On the surface of pure HA sintered at 1250 °C, a new layer precipitated to a smaller extent after 7 days of incubation in SBF compared to the doped samples [95]. Similar results were achieved using an animal model. Kitsugi et al. [96] implanted subcutaneously in male rats two pairs of rectangular samples of the same material: HA sintered at 800 °C or 1000 °C or 1200 °C. Two samples were bound with silk thread and two unbound. The animals were killed and examined 1, 2, and 3 months after surgery. In the case of HA sintered at 800 °C and 1000 °C, the formation of a CaP bonding layer was noted in all samples. In contrast, a layer of CaP between the unbonded samples made of HA sintered at 1200 °C appeared only after 3 months of implantation. Moreover, the formation of a bonding layer was observed only on about half of the contact area of all tested HA samples.

## 7. HA Biocompatibility Dependent on the Sintering Temperature

One of the most important properties of any biomaterial is biocompatibility [12,69,98,99]. This term describes the ability of a material to assist normal cellular activity without inducing undesirable reactions from the host tissues, both locally and systemically [98,100]. Implantation of the material should not contribute to: cytotoxicity, immunogenicity, genotoxicity, mutagenicity, or edema [43]. Another desirable feature of biomaterials used in bone regeneration is osteoconductivity, i.e., the ability to support cell adhesion and proliferation, osteogenic differentiation, and synthesis of the extracellular matrix by osteoblasts on the surface and inside the material structure. The ideal scaffold should further contribute to the formation of new bone tissue through the recruitment and differentiation of progenitor cells and specific molecular signals (osteoinductivity) [100,101]. The impact of sintering temperature on HA biocompatibility is presented in Table 5.

**Figure 4:**
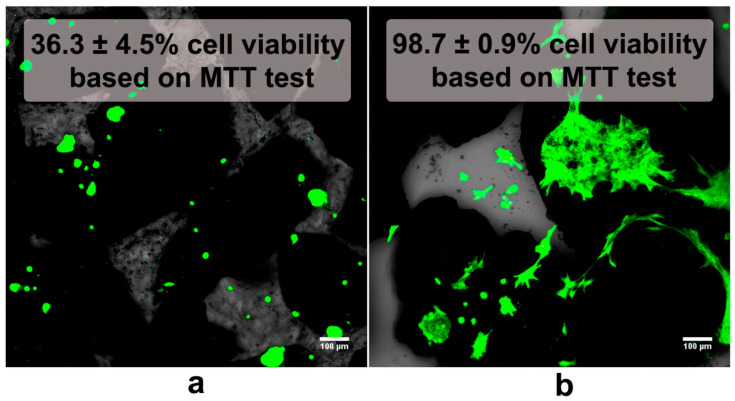
Confocal laser scanning microscope images of osteoblasts after live/dead staining (green florescence—live cells, red fluorescence—nuclei of dead cells) grown on hydroxyapatite (HA) granules sintered at: (**a**) 400 °C (poor adhesion, abnormal morphology, and survivability of the cells) and (**b**) 1000 °C (good adhesion and spreading of the cells, high cell viability).

It should be noted that, according to the available literature, high bioactivity of the HA sintered at low temperatures is directly related to its high SSA and ionic reactivity resulting from the dissolution of bioceramics, ion uptake (often associated with apatite formation), or ionic substitutions. In turn, high ionic reactivity of the HA may significantly change ionic composition in the surrounding microenvironment (especially with respect to Ca^2+^ and HPO_4_^2−^), impairing cell adhesion to the biomaterial and exerting a cytotoxic effect on the bone cells (Figure 4). In general, it was observed that HA sintered at low temperatures causes significant uptake of Ca^2+^ and HPO_4_^2−^ ions from the microenvironment, depriving it from these crucial ions and leading to cell death [34,35,36,37,38]. Laquerriere et al. [102] studied the effect of the sintering temperature of HA on the viability of human monocytes (cell line U-937), which under physiological conditions are one of the first cells to contact the biomaterial after implantation. The results of the research proved that HA sintered at 600 °C had a more toxic effect on monocytes compared to HA sintered at 1180 °C and, interestingly, compared to unsintered HA. It was indicated that the SSA of the ceramics as well as the dissolution and reprecipitation processes (bioactivity) could be responsible for the lack of biocompatibility of the material. Furthermore, Wang et al. [103] assessed the cellular response of SaOS-2 osteoblast-like cells after culture on the surface of sintered HA. Cell proliferation was significantly higher on the surface of HA sintered at high temperature (1200 °C) compared to HA sintered at low temperature (800 °C). In addition, it was observed that the expression of bone sialoprotein (which plays an important role in bone matrix mineralization, calcium and collagen binding, and cell adhesion [106,107]) and osteocalcin (a marker of the late stage of osteoblast maturation [103,108,109,110]) was higher in cells cultured on HA sintered at a high temperature. Another study showed that the number of murine fibroblasts (L929 cell line) decreased over time after their culture in direct contact with HA sintered at lower temperatures (850 °C and 950 °C), while the number of cells cultured on ceramics sintered at 1050 °C (and higher) was constantly increasing. A similar tendency was shown by the results obtained during cell culture without direct contact with the material, which proved that the properties of the culture medium itself changed under the influence of ceramics. In addition, SEM images obtained after 3 days of cell culture on the surface of the material sintered at high temperature revealed a compact layer of well-flattened cells. On the surface of the material sintered at low temperature, however, the cells became rounder, indicating poor adhesion. Remnants of dead cells were also noted. It was also demonstrated that the concentration of phosphate and calcium ions decreased in the culture medium when incubated with HA sintered at low temperature (<1050 °C). Culture with ceramics sintered at a higher temperature resulted in an increase in the concentration of these ions. The authors indicated that the differences in the biocompatibility of ceramics resulted from differences in the SSA, and thus the degree of ion exchange with the culture medium (ionic reactivity) [104]. 

**Figure 5:**
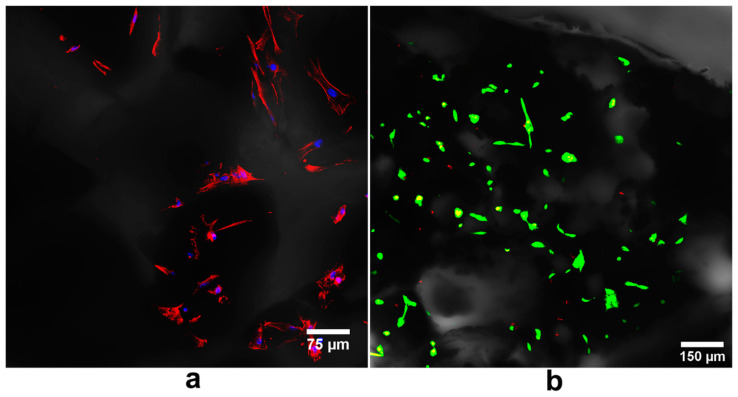
Confocal laser scanning microscope images presenting poor adhesion and abnormal morphology of: (**a**) mouse calvarial preosteoblasts (MC3T3-E1 cells) after cytoskeleton staining (red florescence—cytoskeleton, blue fluorescence—nuclei) and (**b**) human fetal osteoblasts (hFOB 1.19 cells) after live/dead staining (green florescence—live cells, red fluorescence—nuclei of dead cells) grown on the composite biomaterial made of hydroxyapatite (HA) sintered at 800 °C and β-1,3-glucan (unpublished microscope images related to our studies performed previously [34,117]).

**Figure 6:**
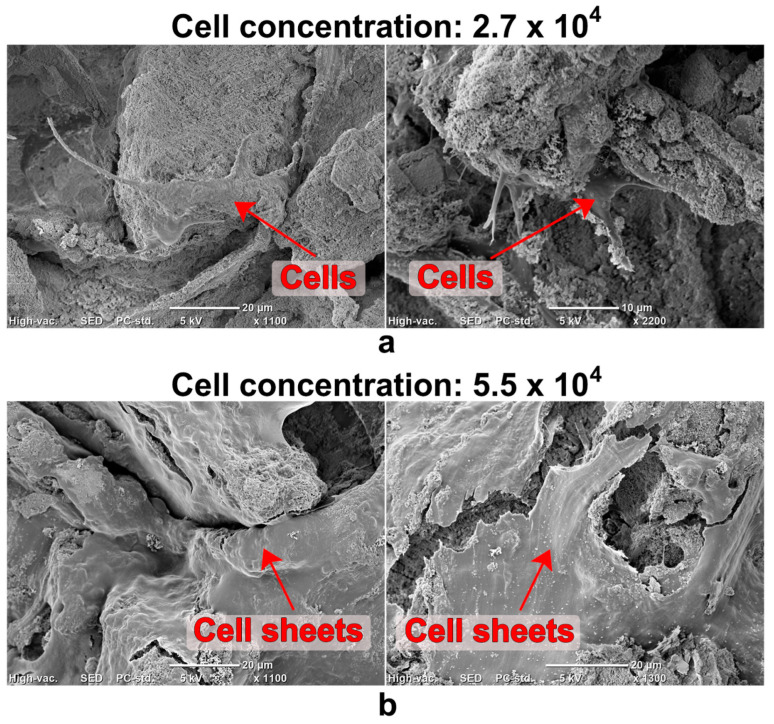
Scanning electron microscope images showing preosteoblasts (red arrows) grown on the composite biomaterial made of hydroxyapatite (HA) sintered at 800 °C and β-1,3-glucan (7 days after cell seeding): (**a**) small number of MC3T3-E1 preosteoblasts on gypsum-free β-1,3-glucan/HA biomaterial, (**b**) excellent cell growth on gypsum-enriched composite that reduced calcium ion uptake by highly reactive HA from the culture medium (unpublished microscope images related to our studies performed previously [111]).

A similar relationship between high ionic reactivity and low biocompatibility was demonstrated by Hyakuna et al. [105]. The results of the study indicated that HA sintered at 1200 °C showed low cytotoxicity, while ceramics sintered at 600 °C and 900 °C were highly toxic to the V79 cell line (Chinese hamster fibroblasts). The degree of toxicity was correlated with the degree of reduction in calcium, phosphate, and albumin in the culture medium. The authors suggested that the toxicity may depend on the level of microporosity, and therefore also the SSA, which was much higher for ceramics sintered at 600 °C and 900 °C than those sintered at 1200 °C. A similar tendency was observed in the case of composite materials. It was proven that HA sintered at low temperature (800 °C) in combination with β-1,3-glucan caused a significant reduction in Ca^2+^ ions in the culture medium, decreasing cell viability to approx. 50% and limiting the growth of mouse calvarial preosteoblasts (MC3T3-E1 cell line) on its surface. Additionally, the observed cells were abnormally flattened (Figure 5) [34]. However, the addition of a gypsum to the β-1,3-glucan/HA composite reduced calcium ion uptake by highly bioactive HA, improving osteoblast growth and proliferation. There were twofold more MC3T3-E1 cells on the surface of the gypsum-enriched biomaterial compared to the gypsum-free one (Figure 6) [111]. Moreover, Malafaya and Reis [35] also noted that composite materials consisting of chitosan, unsintered hydroxyapatite, and glutaraldehyde (as a cross-linking agent) significantly reduced the viability of the murine fibroblasts (L929 cell line). The toxic effects of chitosan and glutaraldehyde were excluded. Then, the effect of unsintered and sintered HA on cell viability was examined. It was proven that the sintered HA did not cause a significant reduction in cell viability compared to the control (even at the highest concentration used: 20%). In contrast, unsintered hydroxyapatite decreased cell viability. A greater decrease in the concentration of calcium and magnesium ions was also demonstrated after 24 h of incubation of the composite material containing unsintered HA in the culture medium.

The surface of the bioceramic has a decisive influence on the degree of exchange between the medium and the material. As the sintering temperature decreases, the degree of porosity and the SSA of the material, which is in contact with the culture medium, increases [8,34,35]. In the case of the presented test results, a large surface of the material correlated with a reduced level of calcium and phosphate ions in the culture medium. These ions are probably used for the formation of apatite on the surface of the ceramic [104]. An appropriate concentration of ions, especially calcium, phosphate, and magnesium, is necessary for cells to function properly. Many types of cells must adhere to surfaces in order to survive. These include osteoblasts, chondrocytes, fibroblasts, and endothelial cells [112]. Cell adhesion receptors, which include integrins, participate in cell adhesion to the substrate as well as to other cells or the extracellular matrix. These receptors have extracellular domains through which they bind to protein ligands. This ligand–receptor binding is dependent on divalent cations, especially magnesium, which are activators of integrins. Therefore, the amount of individual cations (Ca^2+^, Mg^2+^) in the surrounding microenvironment may play an important role in the regulation of cell adhesion [113,114]. Calcium ions also induce the expression of bone-specific proteins, e.g., a bone sialoprotein or osteopontin that is mediated by a special calcium-dependent channel and a kinase [111]. It is also important to maintain the right balance of phosphate ions. It was shown that a deficiency of inorganic phosphate ions reduced the proliferation of mesenchymal stem cells derived from the bone marrow, while their excess induced the process of apoptosis of osteoblasts in vitro [115,116]. 

## 8. Conclusions

The present review article described the process of hydroxyapatite sintering, highlighting the pros and cons of the selected temperature ranges. The variety of studies describing the applied sintering temperature provided valuable scientific knowledge that may be used for HA-based biomaterial synthesis. A high sintering temperature (≥900 °C) resulted in a material with large grains, high crystallinity, low porosity, low SSA, and high biocompatibility. However, this production technique led to the formation of an HA structure with limited clinical applications. The insufficient tensile strength and brittleness of the material, especially in wet conditions, prevented the use of HA in areas requiring load transfer. The negligible biodegradation and bioresorption of HA sintered at high temperatures caused it to remain in the place of implantation for years, hindering the complete regeneration of the bone defect. In addition, the limited bioactivity negatively affected the osseointegration of the material with the host bone. As the sintering temperature decreased, the degree of porosity and the SSA of the HA increased. Importantly, the grain size and crystallinity were reduced compared to HA sintered at higher temperatures. The comprehensive literature review confirmed that the high SSA of the HA material sintered at lower temperatures (<900 °C) directly correlated with its high bioabsorbability as well as its ionic reactivity and thus bioactivity. However, it also caused a significant reduction in the level of calcium, magnesium, and phosphate ions in the culture medium. In turn, the decrease in the concentration of these ions, which are responsible for appropriate cell function, probably contributed to the toxic effects of the HA material sintered at low temperatures. Therefore, it is difficult to determine the ideal synthetic HA produced using the sintering process for biomedical applications. 

To increase the biodegradation rate of HA, biphasic calcium phosphate (BCP), which is the mixture of different concentrations of stable HA and more soluble β-tricalcium phosphate (β-TCP), was introduced in the late 1980s for bone regeneration applications [8,118]. In addition to improved bioabsorbability, the release of controlled levels of calcium ions from BCP over time favors the formation of an apatite layer, providing good bioactivity [118]. Another strategy for obtaining ceramics with high SSA and good bioabsorbability is the production of biomimetic CaPs which closely mimic the composition and structure of bone mineral. These kinds of CaP ceramics can be produced through low-temperature dissolution–precipitation reactions, mimicking the biomineralization phenomena [119]. Nevertheless, the easiest way to produce HA with high microporosity and high SSA is to apply low sintering temperatures. Thus, the continuation of research in this direction is necessary to minimize or overcome the occurrence of side effects related to the mechanical, microstructural, and biological properties of sintered HA. Currently, many areas of research focus on modifications of the properties of HA itself through ionic substitutions and the addition of polymers (production of composite materials) or other ceramic materials.

## Figures and Tables

**Table 1:** Impact of sintering temperature on HA mechanical properties.

Type of Biomaterial	Type of Mechanical Test	Temperature of Sintering	Mechanical Properties		Ref.
			Young’s Modulus (E, GPa)	Compression Strength (MPa)	
HA powder cylindrical specimens	Ultrasonic	1140 °C	16.30	5.26	[44]
		1200 °C	36.30	7.66	
		1300 °C	59.80	13.25	
		1340 °C	73.5	13.81	
HA powder pellets	Universal testing machine (UTM)	900 °C	≈1.6	≈0.39	[24]
		1000 °C	≈3.6	≈0.58	
		1100 °C	≈4.25	≈0.85	
HA powder–sucrose specimens	Nanoindentation	1000 °C	48	-	[45]
		1100 °C	74	-	
		1200 °C	153	-	
		1300 °C	134	-	
		1400 °C	89	-	
HA powder compacts	Universal test machine (DVT.e)	1000 °C	-	18.48	[46]
		1100 °C	-	30.47	
		1200 °C	-	28.85	
		1300 °C	-	29.13	

**Table 2:** Impact of sintering temperature on HA microstructure.

Type of Biomaterial	Temperature of Sintering	Porosity (%)	SSA (m^2^/g)	Density (g/cm^3^)	Ref.
HA powder cylindrical specimens	1140 °C	≈37	-	≈1.6	[44]
	1200 °C	≈23	-	≈2.4	
	1300 °C	≈14	-	≈2.5	
	1340 °C	≈13	-	≈2.6	
HA powder cylindrical samples	1150 °C	-	-	≈1.45	[56]
	1200 °C	-	-	≈2.1	
	1250 °C	-	-	≈2.3	
	1300 °C	-	-	≈2.35	
	1350 °C	-	-	≈2.38	
HA powder cylindrical specimens	800 °C	≈41	-	≈1.15	[18]
	900 °C	≈31	-	≈1.3	
	1000 °C	≈4	-	≈1.8	
	1100 °C	≈4	-	≈2.4	
	1200 °C	≈4	-	≈2.4	
	1300 °C	≈2	-	≈2.5	
	1400 °C	≈2	-	≈2.5	
HA powder pellets	900 °C	51.7 ± 0.06	-	-	[24]
	1000 °C	49.5 ± 0.18	-	-	
	1100 °C	46.9 ± 0.10	-	-	
HA powder compacts	1000 °C	-	-	2.15	[46]
	1100 °C	-	-	2.53	
	1200 °C	-	-	2.93	
	1300 °C	-	-	3.03	
HA powder	550 °C	-	28	-	[57]
	1000 °C	-	3	-	
HA microspheres	500 °C	-	60.44	-	[58]
	600 °C	-	44.13	-	
	800 °C	-	12.45	-	
	1000 °C	-	3.7	-	
	1100 °C	-	2.1	-	
Stoichiometric HA powder	200 °C	-	45.2	-	[59]
	900 °C	-	4.7	-	

**Table 3:** Biodegradation and bioabsorbability of HA materials sintered in a wide range of temperatures.

Type of Biomaterial	Temperature of Sintering	Biodegradation/Bioabsorbability Environment	Duration of the Experiment	Observation	Ref.
Synthetic HA (Calcitite)	Not provided	Tris buffer solution (pH = 7.3) 37 °C	120 h	Poor solubility. The least soluble among the tested apatite materials (Calcitite, BoneSource, Norian cranial repair system (CRS))	[73]
HA scaffold	1250 °C	McIlvaine buffer (pH = 5.5) 37 °C	14 days	Low degradation rate, 3% of mass loss	[74]
HA cylindrical implants	1270 °C	In vivo rabbit model	24 weeks	No significant changes in shape and architecture of the implant	[71]
HA powder specimen	1100 °C 1300 °C	In vivo rabbit model	36 weeks	No signs of bioabsorbability	[75]
HA granules	1150 °C	In vivo rabbit model	12 weeks	No significant changes after implantation period	[76]
HA pellets	1000 °C	In vitro neonatal rabbit osteoclasts	48 h	No signs of material bioresorption	[77]
HA discs	1200 °C	In vitro neonatal rabbit osteoclasts	48 h	No signs of material bioresorption	[78]
Carbonate HA cylindrical implants	1200 °C	In vivo rabbit model	12 months	No signs of bioabsorbability	[79]
HA granules	200 °C 1250 °C	In vivo rat model	3 weeks	Greater number of multinucleated giant cells was observed on HA sintered at 200 °C than on HA sintered at 1250 °C, indicating better bioabsorbability	[80]
HA blocks	900 °C	In vivo human	79 months	HA was replaced by newly formed bone	[81]
HA granules	900 °C	In vivo rat model	6 months	Significant changes in material microstructure indicated its bioabsorbability	[82]

**Table 4:** Impact of sintering temperature on HA bioactivity.

Type of Biomaterial	Temperature of Sintering	Apatite Precipitation Time	Experiment Environment	Observation	Ref.
HA polycrystals	800 °C	9 h	Simulated body fluid (SBF)	Process of apatite formation was slower for HA sintered at 1200 °C compared to HA sintered at 800 °C	[31]
	1200 °C	12 h			
HA granules	800 °C	7 days	Simulated body fluid (SBF)	Higher sintering temperature resulted in prolonged apatite precipitation	[93]
	1200 °C	30 days			
HA powder	200 °C	Up to 24 h	Simulated physiologic solution (SPS)	Apatite layer was formed the fastest on the surface of HA sintered at low temperatures (200 °C and 600 °C)	[59]
	600 °C				
	900 °C				
	1100 °C				
HA powder	1100 °C	7 days	Simulated body fluid (SBF)	Chloride-substituted HA was characterized by higher bioactivity than pure HA powder	[94]
Chloride-substituted HA powder					
HA powder	1250 °C	7 days	Simulated body fluid (SBF)	Pure HA was characterized by smaller newly precipitated apatite layer after 7 days of experiment compared to HA with hexavalent tungsten	[95]
HA with hexavalent tungsten					
HA powder specimen	800 °C	1 month	In vivo rat model	Apatite layer was formed significantly faster on the surface of HA sintered at 800 °C and 1000 °C compared to HA sintered at 1200 °C	[96]
	1000 °C	1 months			
	1200 °C	3 months			

**Table 5:** Impact of sintering temperature on HA biological properties.

Type of Biomaterial	Temperature of Sintering	Experimental Model	Cytotoxicity	Observations	Ref.
HA particles	600 °C	Human monocytic cell line (U-937)	Toxic	High dissolution rate	[102]
	1180 °C		Non-toxic	Low dissolution rate	
HA ceramic discs	800 °C	Human osteoblast-like cells derived from osteosarcoma (SaOS-2)	Non-toxic	Reduced bone sialoprotein and osteocalcin expression, low cell proliferation rate	[103]
	1000 °C			High bone sialoprotein and osteocalcin expression, high cell proliferation rate	
	1200 °C				
HA discs	850 °C	Mouse-established fibroblast cell line (L929)	Non-toxic	Negative cell growth rate	[104]
	1050 °C			Slightly reduced cell growth rate	
	1250 °C			Normal cell growth rate	
HA discs	600 °C	Chinese hamster fibroblast cell line (V79)	Highly toxic	Lack of cell growth on the discs	[105]
	900 °C			Poor cell growth on the discs	
	1200 °C		Low toxicity	Normal cell growth up to 7 days	
Hydroxyapatite-B-1,3-glucan composite	800 °C	Mouse calvarial preosteoblast cell line (MC3T3-E1)	Toxic	High ionic reactivity of the biomaterial, significantly reduced preosteoblast growth on the composite	[34]

## Data Availability

Not applicable.
